# How policymakers innovate around behavioral health: adoption of the New Mexico “No Behavioral Health Cost-Sharing” law

**DOI:** 10.1093/haschl/qxad081

**Published:** 2023-12-06

**Authors:** Samantha J Harris, Ezra Golberstein, Johanna Catherine Maclean, Bradley D Stein, Susan L Ettner, Brendan Saloner

**Affiliations:** Department of Health Policy and Management, Johns Hopkins University Bloomberg School of Public Health, Baltimore, MD 21205, United States; Division of Health Policy and Management, University of Minnesota School of Public Health, Minneapolis, MN 55455, United States; George Mason University Schar School of Policy and Government, Arlington, VA 22201, United States; RAND Corporation, Pittsburgh, PA 15213, United States; Department of Medicine, Division of General Internal Medicine and Health Services Research, University of California Los Angeles, Los Angeles, CA 90095, United States; Department of Health Policy and Management, University of California Los Angeles, Los Angeles, CA 90095, United States; Department of Health Policy and Management, Johns Hopkins University Bloomberg School of Public Health, Baltimore, MD 21205, United States

**Keywords:** behavioral health cost-sharing, Multiple Streams Framework, public policy analysis, access to mental health and substance use treatment, private insurance, MHPAEA, Medicaid Maintenance of Eligibility Public Health Emergency expiration

## Abstract

State policymakers have long sought to improve access to mental health and substance use disorder (MH/SUD) treatment through insurance market reforms. Examining decisions made by innovative policymakers (“policy entrepreneurs”) can inform the potential scope and limits of legislative reform. Beginning in 2022, New Mexico became the first state to eliminate cost-sharing for MH/SUD treatment in private insurance plans subject to state regulation. Based on key informant interviews (*n* = 30), this study recounts the law's passage and intended impact. Key facilitators to the law's passage included receptive leadership, legislative champions with medical and insurance backgrounds, the use of local research evidence, advocate testimony, support from health industry figures, the severity of MH/SUD, and increased attention to MH/SUD during the COVID-19 pandemic. Findings have important implications for states considering similar laws to improve access to MH/SUD treatment.

## Introduction

State health policies can serve as important test cases for advancing novel policy solutions prior to consideration by other state or federal policymakers. Before the 2008 passage of the Mental Health and Addiction Parity Equity Act (MHPAEA), several states, beginning in the 1970s, demonstrated the feasibility and usefulness of mental health parity laws.^1,2^ Groundwork for the Affordable Care Act (ACA) exchanges and Medicaid expansions began with state policy innovation, such as Massachusetts’ 2006 health reform law.^3^

The MHPAEA and the ACA worked together to substantially increase access to comprehensive health insurance that includes coverage of mental health and substance use disorders (hereafter MH/SUD). Nevertheless, privately insured individuals still experience out-of-pocket spending challenges,^4,5^ which have grown with overall increases in commercial insurance plan cost-sharing. These challenges have been especially acute in states such as New Mexico (NM), containing disproportionately large populations of low-income residents and a high burden of MH/SUD.^6–8^ Additionally, while the main impact of MHPAEA and the ACA was improved coverage, MH/SUD service utilization stayed relatively consistent and evidence that parity improved initial access to care or reduced patient out-of-pocket spending is mixed.^8–19^ This could be due to high cost-sharing and quantitative treatment limits (QTLs) and nonquantitative treatment limits (NQTLs), such as medical necessity criteria and prior authorization.^20^ Insurers can require high levels of cost-sharing while remaining parity compliant, and NQTL parity is difficult to enforce.^20^

Motivated by the state's disproportionately high rates of MH/SUD-related morbidity and mortality,^6^ NM lawmakers passed the “No Behavioral Health Cost-Sharing” law (hereafter “NCS”) in April of 2021.^21^ The NCS improves the affordability of care for privately insured New Mexicans who still face high MH/SUD treatment out-of-pocket costs. Out-of-pocket cost is the most commonly cited barrier among patients in need of MH/SUD treatment who do not receive it.^22^ The NCS is the first state law entirely removing in-network MH/SUD treatment copayments, coinsurance, and deductibles in private insurance plans, and is an innovative policy solution to further improve access to care beyond the ACA and MHPAEA regulations.

State health policy entrepreneurs propose potential policy solutions to problems while operating within the policy environment's constraints and opportunities. Prior studies have identified state-level and individual legislator factors that predict state legislator support for comprehensive state parity laws. Such laws are most likely to be approved when parity is in alignment with the political ideology of current leaders and when MH/SUD advocacy organizations are involved, among other factors.^23^ Beyond state-level factors, individual legislator ideology and opinions have been shown to be a large factor in the passage of comprehensive state MH/SUD parity legislation, where beliefs that such laws would increase insurance premiums and MH/SUD stigma can inhibit support.^24,25^

Because the Employee Retirement Income Security Act of 1974 (ERISA) prohibits state regulation of self-insured insurance plans, NM policymakers can only influence insurance plans under the NM Office of Superintendent of Insurance's (OSI) purview, including state and public-school employee health plans and fully insured plans sold on and off the NM Health Insurance Marketplace to individuals, small groups, and to large group employers that are not eligible for marketplace coverage due to size.^26^ The NCS thus excludes self-funded plans. Estimates suggest that two-thirds (66%) of NM residents with employer-sponsored health insurance are enrolled in self-funded plans, which is above the national average (57.9%).^27^ To comply with federal tax regulations, high-deductible health plan (HDHP) enrollees are eligible for no cost-sharing only after their deductible is met.

New Mexico's novel law has important implications for setting policy agendas in other states and federally and can yield important insights for other state and federal policymakers looking for policy options to improve MH/SUD treatment coverage. We share results from a thematic analysis of key informant accounts about how NCS was conceptualized and approved.

## Data and methods

### Data collection

We interviewed legislators, members of OSI who were responsible for law oversight, health care industry leaders including leaders of health plans in the NM marketplace, and MH/SUD and primary care clinicians, many directly involved in the legislative process, about the conceptualization and adoption of NCS (*n* = 30). Table 1 provides participant characteristics. Semi-structured interviews were held over Zoom or phone between February and March of 2022 and averaged 30 minutes. Key informants were recruited by introductions made by NM leaders and through snowball sampling. Interviews were audio-recorded following consent and professionally transcribed.

**Table 1.** Sample characteristics.

ID	Category	Government employee	Role in legislative process
1	State official	Y	Legislator; policy entrepreneur; knowledge broker
2	Insurance leader	N	Knowledge broker; legislator
3	Insurance leader	N	—
4	State official	Y	Expert witness; knowledge broker
5	State official	Y	Knowledge broker
6	Clinician	N	Advocate
7	State official	Y	Legislator
8	State official	Y	—
9	State official	Y	Knowledge broker; legislator
10	Clinician	N	—
11	Clinician	N	—
12	Insurance leader	N	—
13	Clinician	N	—
14	State official	Y	—
15	State official	Y	—
16	State official	Y	—
17	Clinician	N	—
18	Clinician	N	—
19	State official	Y	—
20	State official	Y	Legislator
21	Local official	Y	—
22	Insurance leader	N	—
23	Clinician	N	Advocate
24	Clinician	N	—
25	State official	Y	Insurance regulator
26	State official	Y	Insurance regulator
27	State official	Y	Insurance regulator
28	Clinician	N	—
29	Clinician	N	—
30	Clinician	N	—

Source: Authors’ records of study participants. Many participants had significant prior experience in positions across the health sector. Categories were assigned based on the participant's current role. Participant roles in the legislative process were assigned based on their participation in the law's passage.

Abbreviations: N, no; Y, yes.

We asked participants about NCS's conceptualization, how it gained supporters, how it moved through the legislature, barriers and facilitators to its passage, how it was perceived, and how it was implemented. The current analysis focuses on the passage of the law. The complete interview guide is provided in the Online Appendix.

### Conceptual framework

**Figure 1.**
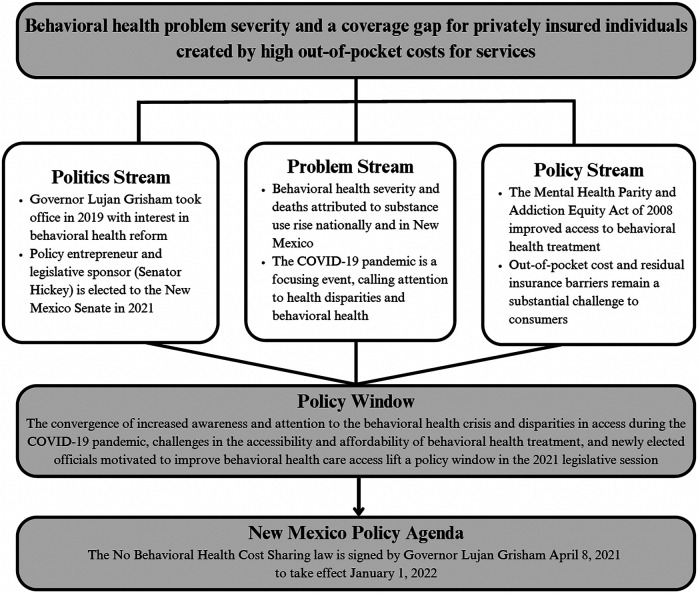
Kingdon's Multiple Streams Framework adapted to the passage of the New Mexico “No Behavioral Health Cost-Sharing” law. Source: Authors’ interpretation of Kingdon's Multiple Streams Framework to understand the No Behavioral Health Cost-Sharing law.^28,29^

We adapted Kingdon's Multiple Streams Framework (MSF)^28,29^ to understand factors leading to the law's passage (Figure 1). The MSF posits that policy changes occur when there is a coupling or convergence between independent streams, including the politics (ie, receptive legislators and policy entrepreneurs that curate policy options within a policy environment's constraints and opportunities), problem (ie, burden of MH/SUD needs), and policy (ie, policy options to address the problem) streams.^29^ Table 2 outlines key MSF components and relevant themes from our analysis.^29,30^

**Table 2.** Operationalizing the Multiple Streams Framework to understand the adoption of New Mexico's No Behavioral Health Cost-Sharing law.

Construct	Relevant themes
Politics stream	
Ideology of legislators	New Democratic leadership Representative bureaucracy Legislature’s motivation to improve behavioral health Lived experiences of legislature
Balance of interests	Perceptions of legislators, industry, and advocates Desire to recover from the behavioral health crisis
Problem stream	
Indicators	Behavioral health morbidity and mortality Cost of behavioral health burden
Focusing event	COVID-19 pandemic
Knowledge broker	The legislative champion/policy entrepreneurs as knowledge brokers Use of local and research evidence
Policy stream	
Value acceptability	Alignment with existing values of policy networks and institutions Alignment with existing policies (MHPAEA and ACA)
Technical feasibility	Demonstration of feasibility in other plans Competing challenges that may not be feasibly addressed or would limit impact (eg, workforce challenges, federal ERISA constraints) Concerns about costs, rates, premiums
Policy community	Role of advocates and expert witnesses Buy-in from health industry
Policy window	
The opening of the policy window	Convergence of the politics, problem, and policy streams open a window of opportunity for policy change

Source: Multiple Streams Framework constructs and themes were adapted from Jones et al.^30^ and Kingdon.^29^

Abbreviations: ACA, Affordable Care Act; ERISA, Employee Retirement Income Security Act of 1974; MHPAEA, Mental Health and Addiction Parity Equity Act.

### Analysis

We analyzed transcripts using ATLAS.ti GmbH version 22 following a hybrid-coding approach,^31^ where an initial codebook was drafted deductively following our study framework and anticipated themes. New codes were created as additional themes emerged. We refined the codebook until the team agreed on the final coding scheme. Transcripts were double-coded until codes were applied consistently and subsequently coded independently. Coded transcripts were reviewed by the study lead. To triangulate findings from the interviews, we reviewed publicly available artifacts from the policymaking process including the law's Fiscal Impact Report, news articles, and legislative session recordings. The Johns Hopkins University Institutional Review Board determined this study to be Not Human Subjects Research.

## Results

### Sample

Key informants (*n* = 30) included 15 state and local officials, 4 leaders from individual carriers with insurance plans with large NM insurance marketplace market shares, and 11 MH/SUD or primary care clinicians (Table 1). Many were involved in the legislative process, as half of the sample were state government employees.

### Politics stream

#### Ideology of legislators: new Democratic leaders, including the central policy entrepreneur, were motivated to reform MH/SUD and respond to previous administration's actions

State governments are dynamic and can face rapid transitions in power, bringing opportunities to implement new agendas. The NCS's champion was Senator Martin Hickey, a newly elected senator who had a prior career as a physician, leader of a managed care organization, and Chief Executive Officer of a large NM insurance plan that previously removed MH/SUD cost-sharing. His health care and insurance expertise afforded credibility when introducing the bill, and many welcomed having clinician representation in the state legislature. As one clinician said, “It’s so good to see Doctor Hickey get voted into the state legislature and to get people in there who have not been part of this whole system for years ….” [Participant 10]. The 2019 election of Democratic Governor Lujan Grisham, an MH/SUD advocate, brought Republican Governor Susana Martinez's 8-year tenure to a close. The elections of Governor Grisham and Senator Hickey ripened NM's political climate for MH/SUD reform.

Their agendas included recovering from the previous administration's perceived harm to the MH/SUD system. In 2013, the NM Human Services Department and then-Governor Martinez alleged Medicaid fraud in 15 MH/SUD agencies,^32^ inducing an MH/SUD crisis after MH/SUD practitioners and organizations left the state, although allegations were later ruled as unfounded and resulted in settlement payments to over half the agencies. The “Great Disaster,” as one called it, “effectively withheld payments and shut down the system, which was disastrous for the network of care and for the service providers. We lost some of our longest term, most prolific and creative service advisors in that period” [Participant 6]. In 2016, then-House Representative Grisham advocated for addressing the crisis in the Congressional Record, calling the MH/SUD system's dismantling “the most egregious abuse of power I have seen in my decades of government service,” and said she “will not sit idly by while the most vulnerable among us suffer.”^33^

#### Balance of interests: the NM legislature and MH/SUD institutions were motivated to address the MH/SUD crisis

Legislators, state officials, and health care industry participants were motivated to address the MH/SUD crisis. The state's ongoing MH/SUD provider shortage, lack of infrastructure, and rurality are known barriers to care. Many felt that NCS was a step in the right direction. One legislator described feeling “hopeful that this [law] will give people the opportunity to get the help they need,” adding that, “We all know if people don’t have insurance or if they have insurance and can’t afford the copay, they will not go. And that's the worst thing we can do for folks.” They hoped NCS could be “one more step towards maybe even just to get back to where we were in 2013 [with the dismantling of the system] and, at some point, go beyond that” [Participant 20]. Another legislator described bipartisan support for MH/SUD, saying, “I think there is bipartisan recognition and support of how devastating the 2013 actions were. I think there's strong recognition across the aisle on not many things, but on this, the need for behavioral health hits so many people …. I think there was pretty good support” [Participant 9]. New Mexico being the first state with a law of this kind was viewed as an exciting opportunity. One state official noted, “I just thought it was kind of like New Mexico doing fantastic in terms of saying, ‘No, we’re not going to limit this (cost-sharing). We’re going to remove the barrier entirely’” [Participant 14].

### Problem stream

#### The state and national mood: the COVID-19 pandemic and increased burden of MH/SUD focused attention to improving MH/SUD treatment access in NM

Many noted that NM's climate was ripe for MH/SUD reform because of MH/SUD conditions’ impact on NM residents.^7^ The COVID-19 pandemic served as a focusing event, garnering support for addressing MH/SUD treatment access. As a clinician said, “There's terrible access. There's a lot of cycling around for patients. COVID has not made things better. The stress of the world has not made things better. Behavioral health problems have not gotten better over the last couple of years” [Participant 29].

Senator Hickey, the key policy entrepreneur, said “timing is everything” when describing his ability to steward the law's passage. He said the increased awareness of MH/SUD challenges during the pandemic facilitated approval, saying, “Nationally we’re paying more attention to mental health than we ever have before. And a lot of that is probably the result of COVID.” He added that the impact of SUD was a main motivation behind the law's conceptualization, and that speaking candidly about the impact of SUD on his family helped garner political will, saying, “The need is there … . It is a very rare family that does not experience a substance use issue within their nuclear family. … Me saying, ‘Hey, you know what, it's all of us [helped]….Let's talk about it’” [Participant 1]. A clinician similarly described that the MH/SUD crisis helped open the policy window, saying, “They’re [the legislature] just fed up. They’ve seen it enough themselves. They’re like, ‘My son has whatever, and the resources are terrible. This is unacceptable.’ And so it kind of took it getting closer to home” [Participant 29].

#### Knowledge brokers: local research evidence from trusted leaders and credible sources facilitated the law's passage

Senator Hickey's health care industry experience facilitated trust. As a clinician-advocate described, “Because of his background and experience also working for a health insurer, he really understands the barriers not just from patient community perspective, but also from health insurance funding [perspective]” [Participant 23]. Similarly, a legislator noted, “It helps immensely that Senator Hickey and Secretary Scrase [Acting Secretary of the Department of Health] used to run HMOs (health maintenance organizations) …. With their history in the field and their assurance and their ability to point to studies that have been done that showed if you don’t put these artificial barriers up, you’ll wind up saving money, I think it made it a little more acceptable” [Participant 7].

### Policy stream

#### NCS was a novel extension of the coverage afforded under MHPAEA and ACA, and in alignment with the values and goals of the legislature and MH/SUD network

The NCS in many ways extended the goals of MHPAEA by controlling consumers’ MH/SUD care costs. One legislator said, “It was our US Senator Pete Domenici, actually, who passed the federal legislation. And so there had been this widespread belief among many of us that the issue had been resolved. And then it turns out not at all” [Participant 7]. Senator Hickey similarly said, “The reality is no one's been enforcing that. It really has had very little application” [Participant 1].

The NCS was designed to advance the access to care goals central to the federal parity law and the ACA. An attorney who worked with the Legislative Finance Committee noted that NCS could serve as protection should the ACA ever be repealed, saying NM was attempting to “put into state law the good parts of the Affordable Care Act so it will survive after a potentially negative ruling from the Supreme Court” [Participant 8].

#### The policy community: nongovernmental expert witness and lobbyist testimony increased law acceptability

The policy community^29,34^ composed of experts and lobbyists facilitated the law's passage. Policymakers in favor of the law contended that it would increase affordability, help families in crisis, and potentially avert downstream costs. One legislator described cost barriers, saying, “If you have to pay copays every time you go for services, people may start, and they don’t finish because they can’t afford to keep it up or they don’t even do it to begin with” [Participant 9]. An insurance leader from the plan that previously removed cost-sharing served as an expert witness; they noted the potential cost-savings, saying, “There should be a financial benefit accrued to the state, to the communities, because people are theoretically getting the better care and better access.” They added, “If people are finally managed in terms of their behavioral health, they’ll do better with their physical health. Cardiac, pulmonary, what have you. So that's the theoretical, and that's the empirically proven, and that hopefully will play out here as well” [Participant 2].

The legislative champion, Senator Hickey, had an important proof-of-concept to sell the plan: as an HMO (health maintenance organization) administrator he had introduced a private insurance plan that included zero cost-sharing for MH/SUD treatment. As a state official tasked with overseeing the law's implementation described, “It may have been that there was at least some demonstration that it didn’t make costs explode for this carrier.” They went on to say this local evidence mollified carrier opposition, adding, "The only opposition that I remember from carriers was from the national AHIP [America's Health Insurance Plans] group. I don’t remember if any of the other carriers did [oppose], but it definitely wasn’t of the nature that this is going to destroy everything. And sometimes we hear that. But on this one, there wasn’t quite as much opposition” [Participant 27]. The insurance leader who served as an expert witness described that, while the national AHIP group opposed, they sided with lobbyists supporting the law, saying, “They [AHIP] were pissed about this. And I was here, and they were there, and tough beans. It was the right thing to do.” They recalled that the state attorney was a “strict textualist” and subsequently, “There was a lot of clarification of language through the 60-day legislative session” [Participant 2].

The MH/SUD advocates, particularly advocates for patients with autism, were critical to the law's passage. New Mexico is a national leader for coverage of applied behavior analysis (ABA), an intensive approach to improving functioning for children with autism that can involve daily specialist sessions. New Mexico passed a 2019 law ensuring ABA coverage in state-regulated plans and Medicaid for patients aging out of the Children's Health Insurance Program plans (which has covered ABA therapy since 2014).^35^ However, several noted affording care was still a challenge for many families.

One leader from the Behavioral Health Providers Association who supported the law shared how these families were among those expected to most benefit from NCS, saying, “When Senator Hickey introduced the bill, it was almost unbelievable. Our biggest participants and supporters were the ABA providers, and they gave compelling testimony about what the cost was to parents and families when they had to pay [copays] for multiple services in a week” [Participant 6]. A clinician who advocated for including ABA noted their appreciation for the “ability to get up close and actually talk eye-to-eye with the policymakers and share our message and have that feel heard” by the legislature, and that “a really critical piece is finding avenues to really have the impact and the message be heard and then, of course, gathering support, which—that's the easy part” [Participant 23].

### Opening of a policy window

With the convergence of the 3 streams, a policy window opened that afforded NCS passage. Participants described NCS passing with relative ease and minimal lobbying efforts, with a participant describing, “It flew through the first two committees very easily. It was very clear that the legislation was well supported” [Participant 23]. The bill was first presented in the House and Senate committees, then to the floor in March 2021. The Democratic majorities in the House and Senate facilitated its passage. Prior to the hearing, the bill received key industry figure support, and Senator Hickey shared materials with committee heads and senate leaders.

Senator Hickey noted his surprise by the level of support the bill received from industry (eg, hospital systems, insurers), saying, “They [industry] actually supported it. They stood up and said we speak for this bill. … To deal with our mental health situation, folks in the industry realize that these small costs upfront have huge benefits, cost wise as well as healthwise, downstream” [Participant 1]. Another legislator recounted the law passing with little or no anticipated insurance industry opposition, saying, “I have to say that we certainly didn’t get enthusiastic endorsement from the insurance industry, but they didn’t fight it tooth and nail because they thought, ‘Well, let's see.’” The NCS sunsets in January 2027, and the legislator predicted, “If the first results aren’t all that positive, we’ll start getting some pushback to change it back. But we can anticipate that it will probably prove our point to them that it does save them money in the long run” [Participant 7]. An OSI official similarly noted, “There was really very little opposition. There was really none. The health insurers didn’t have any strong opposition to it. A couple of them peeped a little that it might increase their premiums” [Participant 26]. A legislator said there should be no impact on premiums, saying, “There's no probably love lost among anyone about insurance companies. So, they need to do what’s right, and this should not impact them negatively. Employers or whoever is purchasing the insurance shouldn’t negatively impact premiums and so forth, because they should see those claims decrease in physical health, particularly ERs and hospitalizations” [Participant 9].

The University of New Mexico's Health Services Center and OSI documented potential concerns in the Fiscal Impact Report^36^ that NCS could cause insurers to decrease provider rates or increase premiums should the initiative not result in cost-savings. On rates, a participant said, “The mix of poverty and the mix of payers in New Mexico, which impacts reimbursement rates, has affected us just like in other states” [Participant 12]. Another explained, “We already have such difficulty in attracting providers to our state. So that is a critical piece of this legislation—that it cannot create more work—non-physician—non-provider tasks, and it cannot adversely affect our reimbursement rates or raise our malpractice” [Participant 17]. The University of New Mexico and General Services Department's Risk Management Division also cited concerns that the impacts of NCS may be limited given that Medicaid, the population with the largest MH/SUD need, has no MH/SUD cost-sharing. The General Services Department's Risk Management Division also noted that state employees have access to 5 free MH/SUD visits per occurrence, per year, and telemedicine at no cost.^36^ Until NCS, the only private insurance plan access to MH/SUD at no cost-sharing was through the health plan the sponsoring Senator previously led.

House Health Committee Representatives had some initial hesitations, questioning why, if parity was the goal, MH/SUD should be at no cost as opposed to all health care. After several hours of House debate, the bill was ultimately “logrolled” with another bill establishing a Health Care Affordability Fund that would pool money to help uninsured New Mexicans enroll on the exchange, a procedure the Legislative Finance Committee documented as potentially unconstitutional under NM law.^36^ The new combined bill had to be presented to the Senate again, where the Head of the Legislative Finance Committee, unconvinced the law would result in savings, voiced concern about costs in the Healthcare Affordability Fund. Ultimately, the Governor signed the bill into law, taking effect January 1, 2022.

While NCS was generally viewed as a positive step, participants noted additional needs to improve the MH/SUD system, particularly if the law had unintended consequences such as increased premiums, reduced provider reimbursement rates, or increased administrative burden. Senator Hickey said the law was a step in addressing shortfalls in the system and toward the enforcement of MHPAEA, concluding that, “Based on this [NCS] bill, I’m realizing, great, we got rid of the financial barriers. The health plans will begin to see the logic. … We are desperate [for more providers]. … So that’s really the next chapter” [Participant 1]. While the Health Care Affordability Fund would help fill coverage gaps in affording premium costs and other copayments, Senator Hickey shared plans for an additional bill (now law, SB273) to enforce MHPAEA and build up the provider network.

## Discussion

New Mexico's NCS law is an innovative strategy for increasing access to MH/SUD care. The NCS was anticipated by OSI to benefit up to 340 000 people enrolled in private insurance plans subject to state regulation. Study participants were mostly in favor of the law and viewed it as a vehicle for improving MH/SUD parity and ensuring NM would retain the protections afforded by MHPAEA and ACA should they be repealed. There was widespread appreciation for the law's novelty and a sense it would provide an important test case for other states.

New Democratic leadership ripened the climate in NM for MH/SUD reform, consistent with prior studies of state legislator support for comprehensive state parity laws, where such laws were more likely to be approved under Democratic leadership.^23–25^ Legislators appreciated the opportunity for NM to be a health policy leader, to improve health equity, and the potential cost-savings, while noting additional efforts were still needed to improve the MH/SUD system and achieve parity. Clinicians and MH/SUD leaders were enthusiastic about improving access to care.

While a promising policy, prior evidence of the impact of MHPAEA on improving access to initial care is limited, although like MHPAEA, the law will likely improve coverage for those currently receiving care. Additionally, it remains to be seen whether NCS has the desired impact of cost-offsets (eg, lower emergency department utilization or cost-savings due to better managed behavioral health), as cost-offsets or savings have not been uniformly observed in evaluations of parity.

Others in our sample and in our review of records expressed concerns about insurance plans raising premium costs or lowering rates for providers, and were not convinced the law would result in savings. Advocates of MH/SUD and individual legislator opinions about NCS played a vital role in the law's passage, consistent with prior research finding that MH/SUD advocacy organization involvement and perceptions that such laws improve access to MH/SUD care while not increasing premiums are important factors in passing comprehensive state-level parity laws.^23–25^ Some were also uncertain whether NCS would have much impact given that New Mexicans enrolled in Medicaid have access to MH/SUD at no cost and state and public employees have access to a limited number of free visits per year.

We examined only the law's passage and anticipated impact. Its legacy will be shaped by subsequent events. For example, the public health emergency order that granted continuous Medicaid enrollment without having to re-verify eligibility expired in 2023. With the “unwinding,” up to 100 000 New Mexicans were anticipated to lose Medicaid and need new coverage.^37,38^ While NCS is not designed to address the unwinding, its importance and political liability will change due to the influx of New Mexicans with low incomes coming into private insurance plans through the exchange who may now have better MH/SUD treatment access.

The NCS law is enforced by NM's OSI. Implementation challenges related to its enforcement should be examined, as prior studies have found that state insurance office enforcement is facilitated by both an alignment with legislator interests and priorities, and positive relationships between insurance offices and groups affected by implementation of parity laws.^39^ The support from the NM governor and sponsoring senator will likely facilitate OSI enforcement. New Mexico should monitor the degree to which insurance carriers and providers adopt NCS in practice, as a prior study documented low fidelity of evidence-based SUD treatment policy uptake when implementing parity policies in state Medicaid programs.^40^ Such monitoring may allow for NM to address shortcomings in response by carriers or providers, and provide support to these organizations to facilitate successful implementation. The MSF could be used in future work to bridge understanding about the law's passage with the law's implementation, enforcement, and outcomes.^41,42^

As with all state insurance laws, the reach of NCS will be limited by ERISA constraints prohibiting the application of state laws to self-insured plans, in which over half of New Mexicans with employer-sponsored insurance are enrolled. Additionally, those enrolled in HDHPs must meet their deductible to be eligible for no cost-sharing, although a prior study found that high deductibles were associated with reduced MH/SUD treatment utilization.^43^ As consumers are aware of NCS, they can self-select into eligible plans and experience the benefit.

Some expressed concern about residual barriers to care imposed by insurer use of QTLs and NQTLs, such as prior authorization requirements or medical necessity criteria. Additionally, NCS only applies to treatment delivered by in-network providers, and the law could have unforeseen impacts on network composition. Patients who see out-of-network providers—likely due to limited in-network provider availability—could potentially be subject to balance billing practices.^44^ New Mexicans may be especially burdened with challenges finding in-network providers to receive the NCS benefit due to the state's workforce limitations. Senator Hickey sponsored the New Mexico Mental Health Parity Act (SB273)^45^ that was signed into law in 2023 that calls on OSI to enforce compliance with MHPAEA, including self-insured plans, and to limit QTL and NQTL use. The new parity law requires plans to increase their rates to grow the workforce.

The NCS will yield important insight for other states looking to limit cost-sharing. Colorado, Connecticut, Delaware, and Massachusetts have passed laws ensuring at least 1 free MH/SUD examination annually,^46^ and Maine has a law that prohibits cost-sharing for up to 3 MH/SUD visits annually. New Mexico is the first to completely eliminate MH/SUD cost-sharing. Future trends will determine the viability of NCS as a long-term solution in NM, and as a policy option for state and federal policymakers.

## Limitations

Our study has limitations. First, responses may have been subject to social desirability bias. Second, our study was conducted approximately 10 months after the law passed. Results may be subject to recall bias. Finally, relying on NM leadership for introductions to participants may have favorably skewed results.

## Conclusion

The NM NCS law is an innovative policy that builds upon the protections afforded by MHPAEA and the ACA's regulations. Against a backdrop of an MH/SUD crisis and under-investment in services, policymakers have a limited set of levers to pull that might have an immediate impact on access to and use of MH/SUD treatment. The NM NCS policy targets 1 lever, cost-sharing, and pushes it further than it has ever been pushed in commercial insurance. The benefits and limits of this policy will be closely watched in NM, and in other states, as state policymakers and advocates consider the next frontier of reforms to MH/SUD financing.

## Supplementary Material

qxad081_Supplementary_Data
