# Supplementary material for: How policymakers innovate around behavioral health: adoption of the New Mexico “No Behavioral Health Cost-Sharing” law
Source: Health Aff Sch. 2023 Dec 6;2(1):qxad081. doi: 10.1093/haschl/qxad081 (PMC10986291; doi:10.1093/haschl/qxad081)
Supplement: qxad081_Supplementary_Data [file qxad081_Supplementary_Data.zip › Appendix.docx]

Online Appendix: Key Informant Interview Guide

Thank you for agreeing to speak with me today about the no behavioral health cost-sharing bill in New Mexico that will eliminate cost-sharing for behavioral health services in private insurance plans. Our team is conducting an evaluation of this novel piece of legislation in hopes of learning how this is implemented so that other states may learn from this policy.

**Section A: Introduction**

First, I’d like to learn about your role and background.

1. What is your current professional role?
2. How long have you held that position?
3. Have you held other positions in your professional career?

**Section B: Legislative Approval Process**

TRANSITION: Thank you. Now I’d like to learn more contextual information about the No Behavioral Health Cost-sharing law. I’d like to ask you to share your current view of the behavioral health landscape in New Mexico, your initial perception of the bill, and your understanding of what it aims to address.

1. First, what is your understanding of how this bill came to be?
   - Probes:
     1. How did you first learn about the behavioral health cost-sharing bill?
     2. What current needs in the state does it intend to address?
     3. Did you have a role in its approval? If so, what was your role?
2. *If applicable,* can you talk me through what you recall about the process by which the bill was approved and the issues that were raised in the discussion of passing it?

- Probes:

1. Barriers and facilitators to approving the bill?
2. Who were the legislation champions?
3. How were barriers/issues resolved?
4. Are those issues still a concern?
5. When you first learned of this effort to remove cost-sharing requirements for behavioral health services, what were your initial thoughts?

- Probes:
  1. Has your initial perception changed?
  2. What have you learned since then?
  3. Where/who did you learn more about the bill from?

1. What do you consider as the central goals of the law, or in other words, what problems does the law intend to address?
   - Probes:
     1. How do you think it will impact the accessibility of behavioral health care?
     2. How do you think it will impact the quality of behavioral health care?
     3. Do you think it will impact groups aside from the privately insured? How so?

**Section C: Implementation Landscape**

TRANSITION: Thank you, that’s helpful for me to know. Now I’d like to ask you a bit more about any barriers and facilitators to implementing the No Behavioral Health Cost-sharing law that you may foresee facing.

1. What is your sense of the level of support for the No Behavioral Health Cost-sharing law—are people generally favorable, or is there opposition?
   - Probes:
     1. What are the positive things people say about the law?
     2. What are the concerns about the law, if any?
     3. How do people in your organization feel?
2. *If applicable:* Has your organization adjusted your plan benefits to meet the requirements of the new law? What was that experience like? How have these changes been communicated to members?
3. What issues, if any, have arisen or might arise in implementing the legislation?
   - Probes:
     1. How will the law be enforced?
     2. *If applicable:* What would be helpful to your organization in addressing these issues? (e.g., education campaign from the state)
     3. Are there enough behavioral health providers in New Mexico to meet the need for behavioral health services?
     4. What would help grow and retain the provider workforce?
     5. Can you describe the process of billing for behavioral services with this new law? Are there things that would further remove barriers to prescribing medications or providing behavioral health services?
4. The No Behavioral Health Cost-sharing law has a sunset date of 2026. What are your thoughts about whether this law will be upheld?
   - Probes:
     1. Is there concern that this law may not be upheld?
     2. What would legislators need to uphold this new cost-sharing provision?

**Section D: Closing**

1. Looking forward, what other needs to improve behavioral health equity are there in New Mexico that may not directly be addressed with this law?
2. Is there anything else about the No Behavioral Health Cost-sharing law that you think is important for me to know that you have not already shared?

That concludes our interview. Thank you again for your time today.
